# Synthesis and Characterization of Optically Transparent and Electrically Conductive Mo-Doped ZnO, F-Doped ZnO, and Mo/F-Codoped ZnO Thin Films via Aerosol-Assisted Chemical Vapor Deposition

**DOI:** 10.1021/acs.cgd.4c01238

**Published:** 2024-12-04

**Authors:** Nan Chen, Iqra Ramzan, Shuhui Li, Claire J. Carmalt

**Affiliations:** Materials Chemistry Center, Department of Chemistry, University College London, 20 Gordon Street, London WC1H 0AJ, U.K.

## Abstract

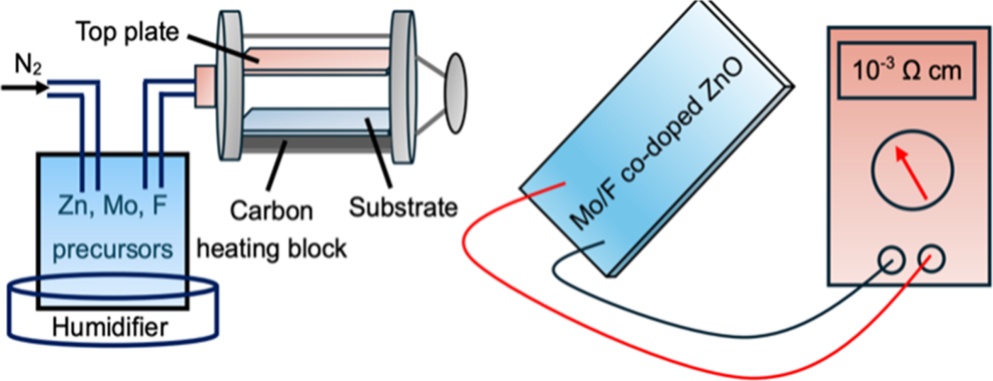

Mo-doped ZnO (MZO), F-doped ZnO (FZO), and Mo/F-codoped ZnO (MFZO) films have been deposited using a simple, cheap, and effective thin-film preparation route, aerosol-assisted chemical vapor deposition (AACVD). ZnO was successfully doped with Mo and/or F, confirmed by X-ray photoelectron spectroscopy (XPS) and by a decrease in unit cell parameters from X-ray diffraction (XRD). XRD also confirmed that all of the films had hexagonal wurtzite ZnO structures. Scanning electron microscopy showed that all of the films had well-defined surface features. The undoped ZnO film had a high resistivity of ∼10^2^ Ω·cm, determined by Hall effect measurements, and a visible light transmittance of 72%, determined by ultraviolet–visible (UV–vis)-IR spectroscopy. The transmittance of the doped and codoped films was improved to 75–85%. The ZnO film codoped with 6.2 atom% Mo and 3.6 atom% F, deposited at 550 °C achieved the minimum resistance (5.084 × 10^–3^ Ω·cm) with a significant improvement in carrier concentration (5.483 × 10^19^ cm^–3^) and mobility (21.78 cm^2^ V^–1^ s^–1^).

## Abstract 2

Mo-doped, F-doped, and Mo/F-codoped ZnO thin films were synthesized using aerosol-assisted chemical vapor deposition (AACVD), a simple and cost-effective method. The films exhibited improved optical and electrical properties and maintained a hexagonal wurtzite structure. This study demonstrates the potential of doping strategies to enhance the performance of ZnO thin films for optoelectronic applications.

## 1 Introduction

Transparent conducting oxides (TCOs) are essential materials in optoelectronic devices such as light-emitting diodes, solar cells, flat screens, touch panel screens, transparent heaters, and photovoltaics.^1−5^ TCOs have metal-like conductivity with a resistivity no more than 10^–3^ Ω·cm and are transparent in the visible region with a transmittance above 80%.^1,4,5^ However, this combination is incompatible based on traditional principles. To achieve transparency, the material must have a large bandgap of >3.1 eV.^4^ The conductivity is a result of the number of free carriers in the system and its mobility.^6^ To achieve conductivity, electrons in the valence band (VB) must absorb energy and thus be excited into the conduction band (CB). The larger the bandgap, the lower the probability that an electron can be excited into the CB, making the pure stoichiometric oxides highly resistive.^3,4,7^ In fact, TCOs can be developed using extrinsic doping to avoid these two mutually exclusive properties, as this strategy allows electron excitation either by a donor level near the CB minimum (n-type) or an acceptor level near the VB maximum (p-type), which improves the electrical properties while maintaining a wide bandgap.^3,4,7,8^

Tin-doped indium oxide (ITO) shows excellent optoelectrical properties, with resistivities of 10^–4^–10^–5^ Ω·cm and visible light transmittance of around 90%, making it the most used TCOs in industry today.^7,9^ However, the market demand for TCOs has continued to grow in recent years, and the low natural reserves of indium have not only led to high costs of this raw material but also increased global competition for controlling the resources, thus restricting indium supply.^3,4^ This is detrimental to countries that completely rely on indium imports. Therefore, it is desirable to develop In-reduced or In-free and earth-abundant TCOs to replace ITO. ZnO is an n-type semiconductor with a hexagonal wurtzite structure. The wide optical bandgap (3.37 eV) of ZnO makes it possible to be a promising TCO material for the next generation as well as its abundant reserves on earth, low-cost, and nontoxicity.^1,2,6^ Doping is required, as pure ZnO shows high resistivity.

Doping of ZnO with Group 13 elements (e.g., Al, Ga, and In) has been widely studied. They form trivalent cations in the ZnO crystal and substitute Zn^2+^, providing an additional free carrier to the system, and hence increasing carrier concentration.^3^ However, doping Al or In in ZnO leads to stability problems because of the ionic radii mismatch between the dopant ions (Al^3+^ 0.53 Å and In^3+^ 0.81 Å) and the host ion (Zn^2+^ 0.74 Å).^6,10^ The formation energy of Al_2_O_3_ (−11.29 eV) is quite low, and hence, there is a high possibility of forming an inactive secondary phase in the resulting films, reducing the electrical conductivity.^11^ Ga and In are more costly in commercial production and Ga is less durable in wet conditions.^12^ Moreover, the resistivity obtained by many research groups is relatively inconsistent (10^–2^–10^–4^ Ω·cm),^3,13−16^ which is mainly due to the scattered mobility values.

The carrier concentration in a semiconductor can be readily varied by doping, whereas the mobility may be reduced through doping.^17^ The system is also negatively affected by free carrier absorption when the carrier concentration is high (>1 × 10^20^ cm^–3^), which can lead to poor transmittance.^18^ Therefore, improving mobility is a key strategy to achieve ideal TCO materials and potential alternatives to ITO.

Recently, transition metal cations (e.g., Mo^4+/6+^, W^4+/5+/6+^, Ti^4+^, Zr^4+^, Hf^4+^, and V^3+/4+^)^1,19−23^ have been employed to improve the conductivity of ZnO. The electrical properties of these materials rely on d-electrons, and hence, they are considered as a new type of TCOs.^24^ They are multielectron donors owing to their larger valence difference between the metal cations and Zn^2+^.^1^ Among them, Mo is stabilized with +4 and +6 oxidation states and could provide the ZnO system with two or four free carriers.^1^ Mo^4+/6+^ (0.65, 0.55 Å) have slightly smaller sizes than Zn^2+^ (0.74 Å), facilitating doping.^14^ Recently, Swallow et al. published a new doping mechanism and applied it to Mo-doped In_2_O_3_ (IMO) deposited via aerosol-assisted chemical vapor deposition (AACVD), which resulted in higher mobility (150 cm^2^ V^–1^ s^–1^) than ITO (80 cm^2^ V^–1^ s^–1^) at the same level of carrier concentration, leading to lower resistivity (∼10^–5^ Ω·cm).^9,25^ Such high mobility is due to the minimum disturbance between the Mo 4d orbitals and In 5s orbital at the CBM.^9,25,26^ This finding indicates that the current electrical performance of TCOs can be obtained by using thinner films, effectively reducing the amount of In needed and thus reducing the cost,^9^ although there are few commercial applications of IMO.^27^ However, this makes Mo a promising dopant and has stimulated interest in the use of this dopant for the doping of earth-abundant ZnO.

The addition of anions (e.g., F and Cl) to ZnO could also enhance the electrical properties of ZnO. They replace O^2–^ sites, providing an extra free electron to the ZnO.^28^ Computational studies have shown that with F-doping, the electronic disturbance occurs mainly in the VB rather than in the CB, which reduces the effect of electron scattering from the CB and hence improves the carrier mobility.^17,28−31^ Moreover, the ionic radius of F^–^ (1.17 Å) is comparable to that of O^2–^ (1.24 Å), thus minimizing lattice distortion.^3,6,17^

So far, numerous studies have been conducted on single-doped ZnO films. Recently, co-doping systems have been developed based on the single-doped ZnO films. This is a new and more complicated class of TCOs that are promising to further advance the functional properties of singly doped ZnO. Cation/cation-codoped ZnO allows each cation in the system to exceed its individual solubility limit, leading to an improvement in the overall dopant solubility of the resulting system, and therefore, achieving a higher carrier concentration than in singly doped ZnO films.^4,17^ In contrast, cation/anion-codoped ZnO aims at providing additional free electron carriers by replacing both Zn and O sites in the crystal with cationic and anionic dopants.^4,17^ In this work, Mo and F were selected as dopants for ZnO, and a codoping system was investigated to further improve the optoelectrical properties of ZnO doped with Mo and F.

ZnO-based TCO films have been synthesized via various routes, such as spray pyrolysis,^28,29^ atomic layer deposition (ALD),^2,17,32^ sol–gel,^33^ magnetron sputtering,^34,35^ pulsed layer deposition (PLD),^36^ and chemical vapor deposition (CVD).^6,7,15,30,31^ This study focused on AACVD. Unlike traditional CVD that requires volatile precursors, precursors for AACVD only need to be soluble in any common organic solvents and can decompose at a given deposition temperature to produce the desired film, allowing for a wider choice of precursors.^7,15,31,37^ Moreover, with this technique, the surface morphology of the films can be readily managed by changing the deposition conditions (e.g., temperatures and solvents), which, in turn, can tune the functional properties.^38−40^ AACVD is a straightforward and effective method that requires only heating of the substrate, requires minimal equipment, can be operated at ambient pressure, and is therefore less costly and suitable for mass production in industries.^1,15^

Here, ZnO, Mo-doped ZnO (MZO), F-doped ZnO (FZO), and Mo/F-codoped ZnO (MFZO) films were synthesized via AACVD. The effects of singly doped ZnO, codoped ZnO, and deposition temperatures on the crystal structure, surface morphology, and the resulting optoelectrical properties were investigated. The formation of the codoped ZnO film offers more possibilities for ZnO-based TCO materials and potential application in future optoelectronic devices.

## 2 Methodology

### 2.1 Film Synthesis

The undoped ZnO, MZO, FZO, and MFZO films were deposited on barrier glass substrates via one-pot AACVD. Zinc acetate dihydrate [Zn(OAc)_2_·2H_2_O], molybdenum hexacarbonyl [Mo(CO)_6_], and ammonium fluoride (NH_4_F) were used as precursors, which were purchased from Merck. Methanol (MeOH) was the only solvent used here. The substrate was treated with a 50 nm SiO_2_ barrier (NSG Pilkington Ltd.) to prevent ion leakage between the substrate and the film. Before deposition, the substrate (15 cm × 4 cm × 0.4 cm) was rinsed sequentially with acetone, detergent, water, and isopropanol.

**Figure 1:**
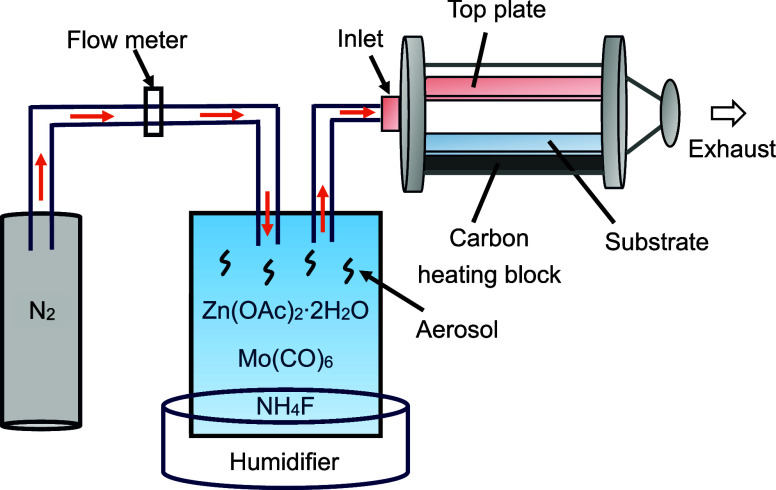
Schematic diagram of AACVD apparatus.

A schematic diagram of the AACVD setup is shown in Figure 1. Zn(OAc)_2_·2H_2_O (0.5 g, 2.28 mmol) in MeOH (25 mL) was used as the precursor for all of the film depositions and was put in a glass bubbler. An ultrasonic humidifier working at 1.6 MHz was used to generate an aerosol from the dissolved precursor solution. N_2_ was then introduced into the precursor solution (flow rate 1.0 L min^–1^), and the aerosol was transported to a preheated glass substrate positioned on a carbon heating block in a quartz tube. A top plate was placed roughly 0.6 cm on top of the substrate to maintain the aerosol flow. For F-doping, NH_4_F (1 mol% with respect to Zn precursor) was dissolved in MeOH and then mixed with the Zn precursor. For Mo doping, Mo(CO)_6_ (3 mol% with respect to Zn precursor) was dissolved in MeOH and then mixed with the Zn precursor. When codoping, Mo(CO)_6_ (2.3 mol % relative to Zn) and NH_4_F (0.75 mol% relative to Zn) were dissolved separately in MeOH and then added to the Zn precursor at the same time. All samples were first deposited at 450 °C, Mo-doped ZnO films were additionally deposited at 500 °C, and Mo/F-codoped films were additionally deposited at 500 and 550 °C.

The precursor–solvent mixture was sonicated for 25 min to give a homogeneous solution before being atomized by using a humidifier. Each deposition lasted (∼35 min) until the precursor solution was used up. Once the deposition was completed, the coated films were cooled to below 85 °C under flowing N_2_ and then removed. The films were cut into 1 × 1 cm^2^ pieces for characterization.

### 2.2 Film Characterization

X-ray diffraction (XRD) was conducted to identify the crystal structure of the films, including preferred orientations, lattice parameters, and crystallinity, and it was carried out by using a Panalytical Empyrean diffractometer with monochromatic Cu Kα_1_ (λ = 1.54056 Å) and Kα_2_ (λ = 1.54439 Å) X-rays. The data were collected over the 2θ range of 10–65° in 0.05° steps of 1 s/step, with an incidence beam angle of 0.5°. X-ray photoelectron spectroscopy (XPS) was performed to determine the chemical compositions, their oxidation states, and dopant concentrations of the films, and it was carried out by using a Thermo Scientific Kα spectrometer with a monochromatic Al–Kα radiation source. Higher resolution scans were collected for the principal peaks of C(1s), Zn (2p), Mo (3d), F (1s), and O (1s) regions at a pass energy of 50 eV. Peak fitting was carried out by using Avantange software, and the binding energies were calibrated to C 1s at 284.5 eV. A scanning electron microscope (SEM) was used to study the surface morphology of the films, and the measurements were performed using a JEOL JSM-6301F field emission SEM at an accelerating voltage of 10.0 kV. All of the samples were gold-coated before measurement. Hall effect measurements were used to determine the electrical parameters of each film, which included resistivity, carrier concentration, and mobility, and the measurements were conducted using the van Der Pauw method. The optical properties, involving transmittance and reflectance, of each film were evaluated in the wavelength of 300–2500 nm using a Shimadzu 3600i plus UV–vis-IR spectrophotometer.

## 3 Results and Discussion

### 3.1 Film Synthesis

The pure ZnO film was prepared from zinc acetate dihydrate with a methanol aerosol. The doped and codoped ZnO films were produced by adding dopant amounts of molybdenum hexacarbonyl and/or ammonium fluoride in methanol to the zinc acetate dihydrate solution.

Here, Zn(OAc)_2_·2H_2_O was used as a host precursor, which is a low-cost, low-toxicity, air-stable, and easy-to-handle compound. For AACVD, there are other commonly available Zn precursors, such as zinc acetylacetonate, which is easily hydrolyzed and has solubility problems at neutral pH,^31^ and diethyl zinc, which is highly pyrophoric and dangerous in both laboratory and industry use.^15^ In addition, a one-pot AACVD was performed in which all of the precursors were dissolved in one solvent (MeOH) and then aerosolized from one glass bubbler. This method is highly suited to generate aerosols that are well-mixed and have a controlled chemical content.^7^

The films discussed here have the lowest resistivity that could be achieved after trying a range of doping concentrations at the corresponding deposition temperature; see the Supporting Information for more details. The adherence of each film to the glass substrate was high, passing the Scotch tape test. The doped and codoped films were visually transparent but showed some colored interferences if viewed from certain angles, which was attributed to the reflections at the three boundaries: air, film, and barrier glass substrate.^10,17,18^ The functional properties of the films did not degrade after dipping them in common solvents, e.g., acetone and methanol, indicating good stability.

### 3.2 Elemental Analysis

The chemical compositions and their oxidation states were determined by XPS. Table 1 summarizes the doping concentrations in the precursor solution (mol%) and the actual doping concentration (atom%) of the successful incorporation into ZnO. These doping concentrations yielded the optimal conductivity at the corresponding deposition temperature, and unless otherwise indicated, the films are labeled with the deposition temperature for the discussion herein. In this study, the dopant(s) atom% were higher than the mol%, which could be due to the dopant being surface segregated. In previous studies, surface segregation was commonly observed in doped ZnO films (e.g., with P, Si, and Al as dopants) deposited via AACVD.^15,37,40,41^ Other factors that may contribute to this discrepancy include variations in the dopant incorporation efficiency across aerosol droplets and differences in precursor volatilities during deposition. While AACVD generally improves dopant distribution compared to traditional CVD, these factors can still lead to deviations in dopant concentration in the film from the initial solution ratios.

**Table 1:** Doping Concentrations of the MZO, FZO, and MFZO Films in mol% and atom%^a^

film	tenperature (°C)	Mo mol%	Mo atom%	F mol%	F atom%
ZnO	450				
FZO	450			1	1.52
MZO	450	3	4.6		
MZO	500	3	8.2		
MFZO	450	2.3	5.7	0.75	1.29
MFZO	500	2.3	5.8	0.75	2.08
MFZO	550	2.3	6.2	0.75	3.64

a Mol% is the dopant concentration in the precursor solution, while atom% is the dopant content in the film, as determined by XPS.

**Figure 2:**
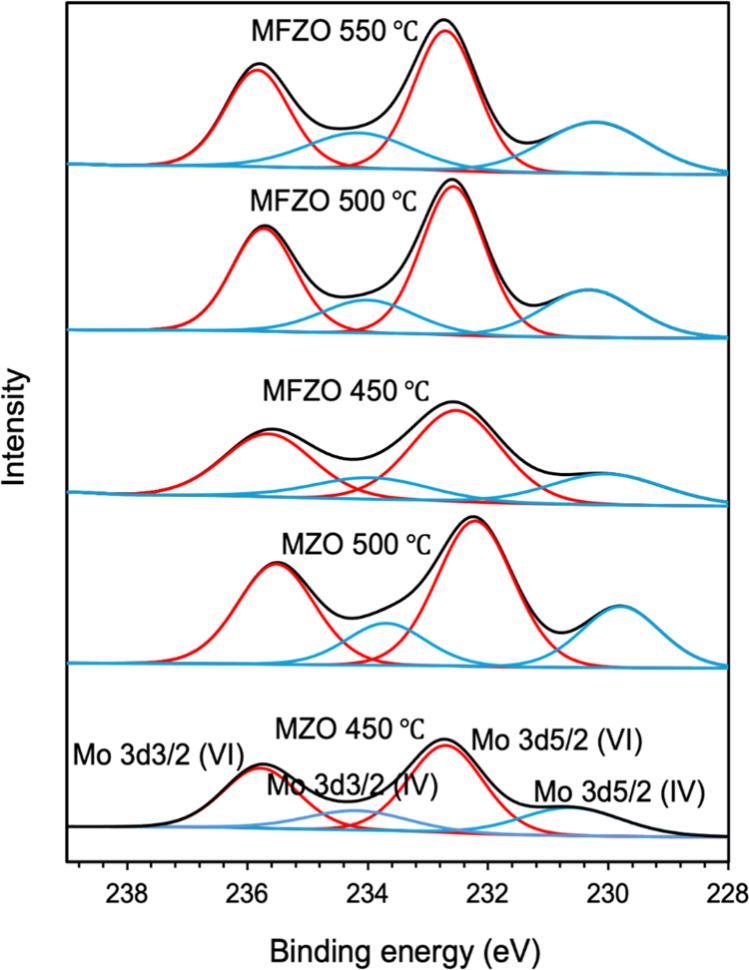
High-resolution XPS spectra of Mo 3d of the ZnO, FZO MZO, and MZO films.

**Figure 3:**
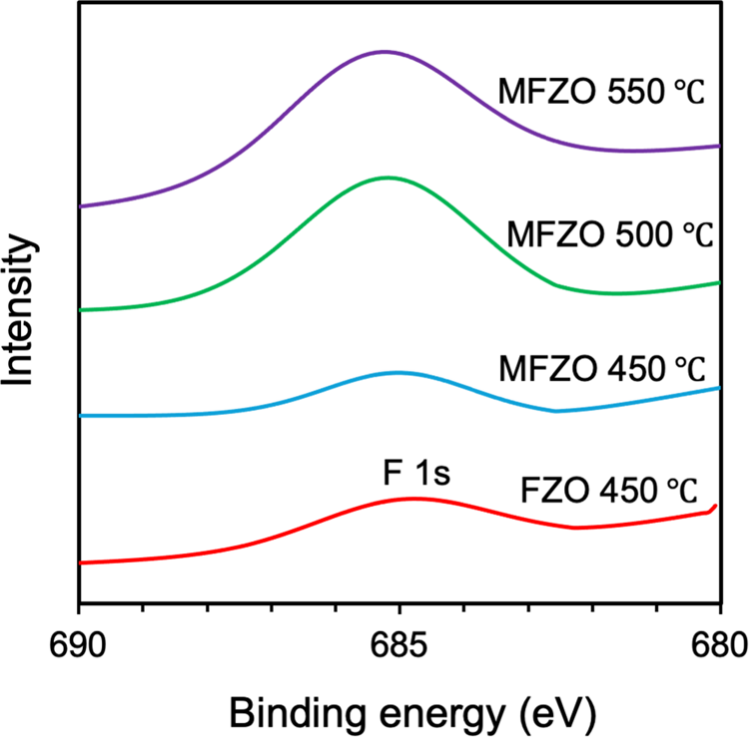
High-resolution XPS spectra of F 1s of the ZnO, FZO MZO, and MZO films.

**Figure 4:**
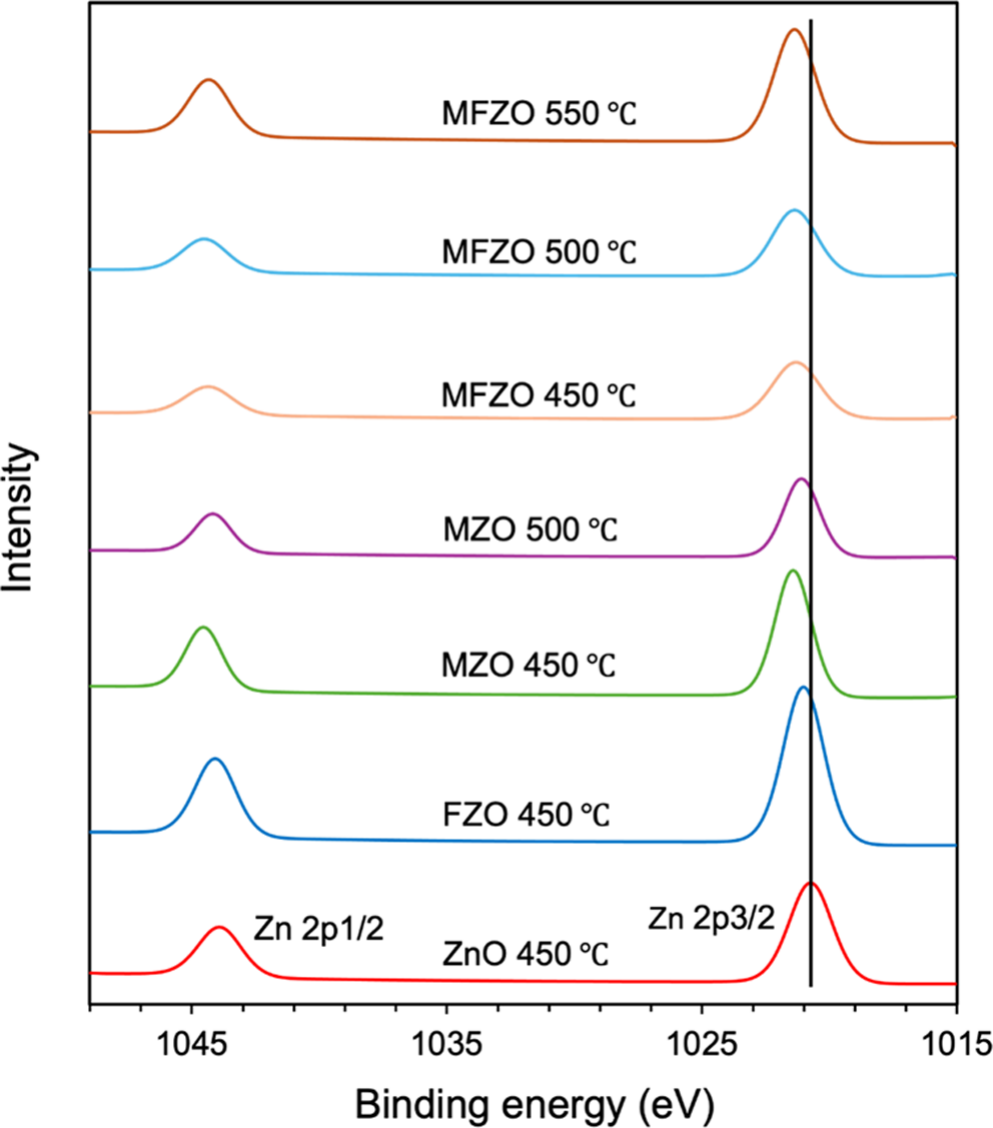
High-resolution XPS spectra of Zn 2p of the ZnO, FZO MZO, and MZO films.

Figures 2, 3, and 4 show the high-resolution XPS spectra of the Mo (3d), F (1s), and Zn (2p) peaks, respectively. For the films containing Mo, two doublet peaks corresponding to Mo 3d can be observed after peak fitting, which were assigned to Mo–O bonds (Figure 2), indicating the presence of two oxidation states of Mo in ZnO. The peak at 232.5 (±0.3) eV and 235.7 (±0.2) eV corresponds to Mo(VI) 3d_5/2_ and Mo(VI) 3d_3/2_, respectively, while the peak at 230.2 (±0.4) eV and 234.0 (±0.3) eV corresponds to Mo(IV) 3d_5/2_ and Mo(IV) 3d_3/2_, respectively.^1^ For the films containing F, the F 1s peak was detected with a binding energy of 685.0 (±0.3) eV and was assigned to Zn–F (Figure 3).^36,42,43^ This signal was weaker than those for Zn 2p and Mo 3d, as the F element is light and difficult to detect.

For the ZnO film, the peak at 1020.7 and 1043.9 eV corresponds to Zn(II) 2p_3/2_ and Zn(II) 2p_1/2_, respectively, which was assigned to Zn–O (Figure 4).^30,31^ However, for the doped and codoped ZnO films, the Zn(II) 2p_3/2_ peak position slightly shifted to higher binding energies, with values between 1021.1 and 1021.4 eV. This variation can be explained by the Pauling electronegativity principle.^44^

**Figure 5:**
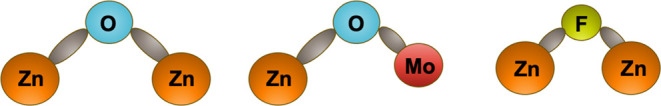
Bonding diagrams of Zn–O–Zn, Mo–O–Zn, and Zn–F–Zn, demonstrating how electronegativity differences affect bond lengths.

Figure 5 shows the bonding diagram of Zn–O–Zn, Mo–O–Zn, and Zn–F–Zn. The bond lengths of Zn–O, Mo–O, and Zn–F are 1.876, 1.730, and 1.763 Å, respectively.^45,46^ Since Mo is more electronegative than Zn, the electrons in the Zn–O–Mo bond are pulled toward Mo, leading to a higher binding energy for Zn 2p. Similarly, F is more electronegative than Zn, and thus, the electrons in the Zn–F bond are pulled toward F, increasing the binding energy of Zn 2p.

The oxidation state of molybdenum in the bulk was unknown as the current study did not use hard XPS for further characterization. However, previous computational studies by Bhachu et al. and Swallow et al. have shown that Mo^4+^ is most favorable in the bulk for Mo-doped In_2_O_3_ films based on density functional theory calculations.^9,25^ In addition, the ionic radius of Mo^4+^ (0.65 Å) is closer to that of Zn^2+^ (0.74 Å) compared to that of Mo^6+^ (0.55 Å). Therefore, it is reasonable to deduce that Mo^4+^ is more likely to be found in the bulk of the ZnO lattice to minimize lattice disorder. The appearance of Mo^6+^ on the lattice surface could be attributed to surface oxidation.

### 3.3 Crystal Structure

**Figure 6:**
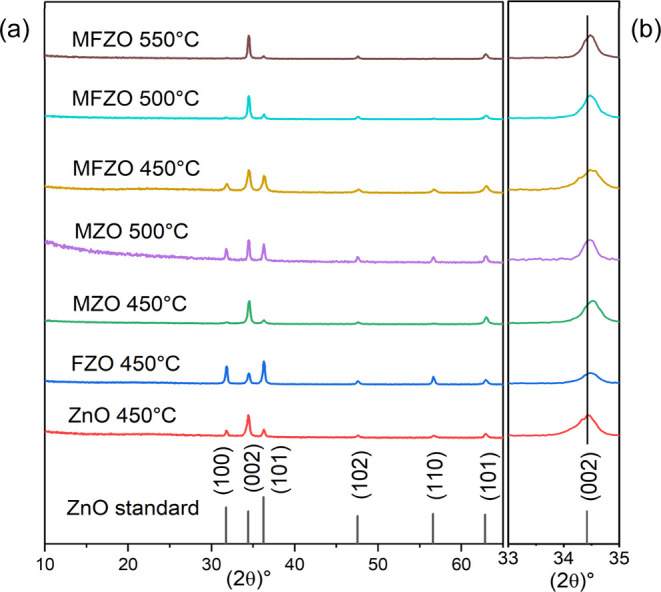
(a) XRD spectra of the ZnO, FZO MZO, and MFZO films. ZnO standard is included for reference. (b) Image on the right shows the magnified (002) peak.

The crystal structure of the films was studied using XRD, which included preferred orientation, unit cell parameters, and crystallite size. First, all of the films exhibited hexagonal wurtzite structure characteristic of ZnO (Figure 6a). No additional peaks corresponding to Mo and F and their oxide secondary phases were detected in the XRD of the doped and codoped films.

### 3.4 Preferred Orientation

The preferred orientation of the ZnO-based films is dependent on the crystal surface energy and can be determined by texture coefficients (TC), eq 1.
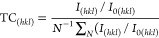
1*I*_(*hkl*)_ is the measured intensity of (*hkl*) plane, *I*_0(*hkl*)_ is the standard intensity of (*hkl*) plane, and *N* is the number of reflections in XRD spectra.^42^ Here, the TC_(*hkl*)_ of the undoped ZnO film was determined by comparing the peak intensities of the undoped ZnO film with those of the standard ZnO, while the TC_(*hkl*)_ of the doped and codoped films was determined by comparing with those of the undoped ZnO film. A summary of TC_(*hkl*)_ is given in Table 2. TC_(*hkl*)_ greater than one means that the plane is preferred, and vice versa.

**Table 2:** Texture Coefficients (TC) of the ZnO, MZO, FZO, and MFZO Films

film	temperature (°C)	TC (100)	TC (002)	TC (101)	TC (102)	TC (110)	TC (103)
ZnO	450	0.68	2.80	0.44	0.72	0.47	0.89
FZO	450	1.33	0.28	1.45	0.70	1.65	0.59
MZO	450	0.40	1.35	0.65	0.99	0.50	2.11
MZO	500	1.00	0.53	1.13	1.23	1.26	0.85
MFZO	450	0.86	0.71	1.37	0.90	1.03	1.13
MFZO	500	0.40	1.57	0.90	1.34	0.53	1.27
MFZO	550	0.23	1.63	0.53	1.56	0.33	1.71

This work revealed that the preferred orientation of the ZnO-based films was affected by the dopants. The undoped ZnO film was observed to be (002)-oriented. At the same deposition temperature, the preferred orientation remained in the (002) plane after doping with Mo but started to grow in the (103) plane. Upon F-doping, the growth in the (002) plane was suppressed and replaced by preferential growth toward the (100), (101), and (110) planes. For the MFZO film, the (101), (110), and (103) planes were preferred, and it is worth noting that the TC values of each plane lie between those of the MZO and FZO films.

Deposition temperature is also a potential factor in the resulting preferred orientation. The preferred orientations of the MZO films varied from the (002) and (103) planes to the (100), (101), (102) and (110) planes as the temperature increased from 450 to 500 °C. For MFZO films, the preferred orientation changed to the (002), (102) and (103) planes with increasing temperatures. Moreover, the codoped films deposited at 500 and 550 °C showed the same preferred growth, but the growth orientation of these planes was enhanced at a higher temperature.

The (002) plane is the most reported preferred orientation in previous studies as it has the lowest surface energy in ZnO,^7,11,37,43^ but this work showed that dopants and deposition temperatures can lead to a change in preferred orientation. In some cases, nonpolar plane growth is also preferred, which usually occurs when different Zn precursors are used. For example, several studies have reported that ZnO films deposited using oxygen-deficient precursors (e.g., ZnEt_3_) preferred to grow in the (101) and (110) planes.^1,6,39^

### 3.5 Unit Cell Parameters

The lattice parameters *a* and *c* of the ZnO, MZO, FZO, and MFZO films, as well as their unit cell volumes were calculated based on eqs 2 and 3, and the data is summarized in Table 3.
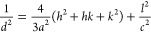
2
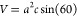
3

**Table 3:** Lattice Parameters, Unit Cell Volume, and Crystallite Size of the ZnO, MZO, FZO, and MFZO Films

film	temperature (°C)	*a* (Å)	*c* (Å)	volume (Å^3^)	volume contraction (%)	crystallite size (Å)
ZnO	450	3.2499	5.2092	47.648		193.73
FZO	450	3.2401	5.1958	47.239	0.86	267.51
MZO	450	3.2432	5.1938	47.311	0.71	223.89
MZO	500	3.2484	5.2004	47.523	0.26	330.30
MFZO	450	3.2404	5.1982	47.271	0.79	168.89
MFZO	500	3.2510	5.1978	47.576	0.15	265.43
MFZO	550	3.2379	5.1994	47.207	0.92	302.44

The lattice parameter along the *c*-axis decreased from undoped to doped/codoped ZnO films, leading to volume contraction. This finding was expected as the ionic radii of Mo^4+^ (0.65 Å), Mo^6+^ (0.55 Å), and F^–^ (1.17 Å) are smaller than those of Zn^2+^ (0.74 Å) and O^2–^ (1.24 Å), which confirms the successful incorporation of Mo and F into ZnO lattice. Similar findings have been reported in Si-doped ZnO,^37^ Al-doped ZnO,^15^ Ga-doped ZnO,^47^ B-doped ZnO,^48^ W-doped SnO_2_,^27^ and Al/F-codoped ZnO films,^6^ where the ionic radii of these dopants are smaller than those of the host ions. In this work, the percentage of volume contraction varied from 0.15 to 0.92. This variation could be due to the presence of varying degrees of electronic repulsion in the lattice. The substitution of Zn^2+^/O^2–^ by Mo^4+/6+^/F^–^ resulted in an increase in free electrons due to the valence difference and led to varying degrees of electronic interactions in ZnO. However, for all of the films, this repulsion did not outweigh the volume contraction caused by smaller dopant ion replacement.

The replacement of Zn^2+^ and O^2–^ sites by the smaller size of the dopants also led to crystallinity changes, eventually causing the peak position of the (002) plane to slightly shift to the right (Figure 6b). Based on Bragg’s law
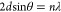
4where *d* is the spacing between the diffraction planes, θ is the diffraction angle, and λ is the wavelength of X-rays.^47^ The larger diffraction angle results in smaller spacing between planes, which suggests that the crystal structure of the films becomes more closely packed after doping Mo or F in ZnO.

### 3.6 Crystallite Size

The crystallite size of each film was calculated by the Scherrer equation
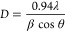
5where *D* is the crystallite size, λ is the wavelength, β is the full width at maximum (fwhm), and θ is the diffraction angle.^42^ As shown in Table 3, the crystallite size of the doped and codoped ZnO films was generally larger than that of the undoped ZnO, suggesting that doping Mo and/or F in ZnO could improve the crystal growth quality. This finding was desired for TCO applications as it can mitigate the scattering effects in the lattice and hence improve the optoelectrical properties.^31,37^ There was no significant correlation between the crystallite size and dopants, but it was affected by deposition temperatures. The crystallite size of the MZO films improved from 223.89 to 330.30 Å as the temperature was increased from 450 to 500 °C while keeping the dopant concentration in the precursor solution constant. Similarly, the crystallite size of the MFZO film improved from 168.89 to 302.44 Å as the temperature was increased from 450 to 550 °C. These results indicated that increasing deposition temperatures led to a larger grain size and thus higher crystallinity, which was consistent with SEM studies. This can be explained by the fact that both the substrate and the particles would gain more energy as the temperature increases, which enhances the diffusion ability of the adsorbed species and thus adjacent particles can combine into larger particles.^36,49,50^

### 3.7 Surface Morphology

**Figure 7:**
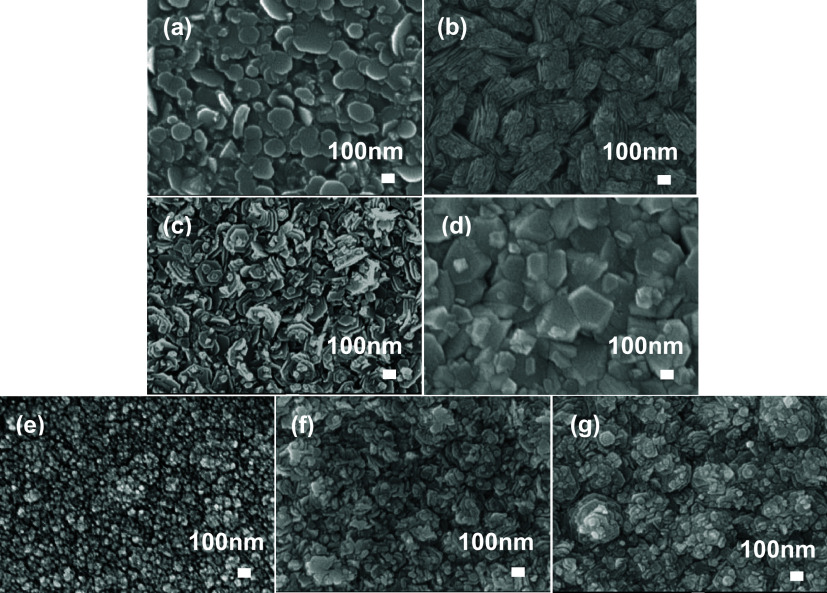
SEM images of (a) ZnO deposited at 450 °C, (b) FZO (1 mol % F) deposited at 450 °C, (c) MZO (3 mol % Mo) deposited at 450 °C, (d) MZO (3 mol % Mo) deposited at 500 °C, (e) MFZO (2.3 mol % Mo + 0.75 mol % F) deposited at 450 °C, (f) MFZO (2.3 mol % Mo + 0.75 mol % F) deposited at 500 °C, and (g) MFZO (2.3 mol % Mo + 0.75 mol % F) deposited at 550 °C.

The surface morphology of the ZnO-based films was determined by SEM, and it is shown in Figure 7. All of the films displayed well-defined structures.

The undoped ZnO film deposited at 450 °C exhibited plate-like structures parallel to the substrate (Figure 7a), which was supported by the preferred orientation along the *c*-axis (002) plane observed from the XRD and in agreement with literature.^39^ Under the same deposition conditions, the film showed petal-shaped features after Mo doping (Figure 7c), while the film exhibited striped-like grains upon doping with F (Figure 7b), and the codoping film became grainy and flatter (Figure 7e). These findings indicated that the surface morphology of the ZnO-based films was highly dopant dependent. This work also showed that the film morphology was controlled by deposition temperatures. It was observed that the morphology of the MZO film changed from petal-shape to columnar (Figure 7d) as the deposition temperature raised from 450 to 500 °C while keeping the dopant concentration in the precursor solution constant, and the crystallite size also increased. The surface morphology of the MFZO film showed some layered features when the temperature was raised (Figure 7f,g), which was the most obvious at 550 °C. This grain growth mechanism is uncommon in ZnO-based films. Moreover, the gradual increase in the grain size was supported by XRD (Table 3).

In comparison to the surface morphology obtained by other deposition routes, Ravichandran et al. found that the ZnO, MZO, and MFZO films prepared by spray pyrolysis deposition did not show significant differences in morphology, with all of the films showing hexagonal plate-like structures.^42^ In contrast, the easily controlled surface morphology is one of the advantages of using the AACVD technique, which, in turn, can tune the functional properties. In addition to dopants and substrate temperatures reported in this work, the morphology of ZnO can show different surface features when using different precursors, solvents, or even adding a few drops of additives.^38−40^

### 3.8 Electrical Properties

The electrical properties of the ZnO, MZO, FZO and MFZO films were determined by Hall effect measurements, and their electrical parameters are summarized in Table 4. The undoped ZnO film showed a high resistivity of ∼10^2^ Ω·cm with a low carrier concentration of ∼10^14^ cm^–3^. Upon doping with Mo or F, an increased carrier concentration is expected, which can be explained by the valence difference between the dopants and host ions, as it brings extra free electrons to the conduction band. This work showed that the MZO and FZO films deposited under the same conditions displayed different electrical properties. The doping concentration was optimized to 4.6 atom % Mo and 1.5 atom % F (Table 1) for both films, as deposited at 450 °C. The MZO film showed a resistivity of 1.81 × 10^–1^ Ω·cm with relatively high carrier concentration (8.61 × 10^18^ cm^–3^) but low carrier mobility (4.00 cm^2^ V^–1^ s^–1^). In contrast, the FZO film showed a resistivity of 4.52 × 10^–1^ Ω·cm with relatively high carrier mobility (25.54 cm^2^ V^–1^ s^–1^) but low carrier concentration (5.41 × 10^17^ cm^–3^). Such differences in the order of magnitude of the electrical parameters were caused by the dopant and its concentration. Mo is a high-valence dopant that replaces one Zn site in the lattice to yield more than one electron, while F is a one-electron donor dopant. From XPS data (Table 1), the MZO film contained a higher dopant amount than the FZO film. The higher the doping level, the more host ions replaced and the higher the carrier concentration. However, these Mo impurities in the lattice were also scattered centers.^37^ The increased number of free electrons also led to high electron–electron scattering.^36^ In addition, the grain size of the MZO film (223.89 Å) was found to be relatively small compared to that of the FZO film (267.51 Å) (Table 3), which led to higher grain boundary scattering, thus reducing the mobility. These scattering effects caused relatively low carrier mobility for the MZO film. Based on the computational studies, the high mobility of the FZO film could also be the result of less electronic disturbance in the conduction band.^17,28−31^

**Table 4:** Optoelectronic Parameters of the ZnO, MZO, FZO, and MFZO Films^a^

film	temperature (°C)	*T*_λ400–700_ (%)	*E*_g_ (eV)	N (cm^–3^)	μ (cm^2^ V^–1^ s^–1^)	ρ (Ω·cm)
ZnO	450	72	3.27	2.94 × 10^14^	12.13	9.53 × 10^2^
FZO	450	85	3.31	5.41 × 10^17^	25.54	4.52 × 10^–1^
MZO	450	77	3.26	8.61 × 10^18^	4.01	1.81 × 10^–1^
MZO	500	75	3.31	1.25 × 10^19^	7.94	6.30 × 10^–2^
MFZO	450	83	3.29	1.18 × 10^19^	8.68	6.10 × 10^–2^
MFZO	500	82	3.30	2.58 × 10^19^	16.79	2.51 × 10^–2^
MFZO	550	79	3.29	5.48 × 10^19^	21.78	5.08 × 10^–3^

a *T*_λ400–700_: average transmittance between 400 and 700 nm; *E*_g_: bandgap; *N*: carrier concentration; μ: carrier mobility; ρ: resistivity.

Compared to IMO, this work did not achieve a high carrier mobility when Mo was introduced in ZnO, but it showed the benefit of F-doping in increasing the carrier mobility. Both the MZO and FZO films showed the same order of magnitude of resistance (∼10^–1^ Ω·cm) but with different orders of magnitude of carrier concentration and mobility, indicating that a balance between these two electrical parameters is essential to achieve a minimum resistivity.

The simultaneous introduction of Mo and F into ZnO further improved the film conductivity under the same deposition conditions. In this case, the doping concentration was optimized at 5.7 atom% Mo and 1.3 atom% F (Table 1), and a lower resistivity value (6.10 × 10^–2^ Ω·cm) was obtained. Since cation/anion-codoped ZnO aims at obtaining free carriers by replacing both Zn and O sites in the crystal with cationic and anionic dopants,^4,17^ it was expected to achieve higher carrier concentration (1.18 × 10^19^ cm^–3^). In addition, there was a slight increase in mobility (8.68 cm^2^ V^–1^ s^–1^) compared to that of the MZO film.

This work also revealed that varying the deposition temperature resulted in changes in the electrical parameters of the ZnO-based films. For singly doped ZnO films, the resistance of the MZO film (6.30 × 10^–2^ Ω·cm) decreased considerably when the temperature was raised to 500 °C. This improvement was attributed to simultaneously increasing the carrier concentration and mobility. First, Mo ions gained more energy at a higher temperature, leading to more Mo ions successfully replacing Zn ions in the lattice, thus increasing the number of free electrons. This result was confirmed by XPS data, which showed 4.6 atom% Mo in the ZnO when deposited at 450 °C, and 8.2 atom% Mo when deposited at 500 °C (Table 1). Second, the crystallinity was improved at 500 °C, resulting in a larger crystallite size, thereby reducing grain boundary scattering and ultimately improving the carrier mobility. This result was confirmed by XRD and SEM studies, in which the grain size increased from 223.89 to 330.30 Å (Table 3) when the deposition temperature was increased to 500 °C.

For codoped ZnO films, the electrical conductivity was also enhanced when raising the substrate temperatures. The cause for the increase in carrier concentration in the codoped films is similar to the results for the increased deposition temperature of the MZO films. A more important finding is the significant increase in carrier mobility of the MFZO film deposited at 500 °C (16.79 cm^2^ V^–1^ s^–1^), which was roughly twice the mobility of the MFZO film deposited at 450 °C (8.68 cm^2^ V^–1^ s^–1^) and the MZO film deposited at 500 °C (7.94 cm^2^ V^–1^ s^–1^). Moreover, as the deposition temperature was raised to 550 °C, the resistance of the MFZO film was further reduced to 5.084 × 10^–3^ Ω·cm and the mobility increased to 21.78 cm^2^ V^–1^ s^–1^. The increasing mobility could be due to increased crystallite size which reduced the scattering effect, codoping of Mo and F achieving a reduction in the grain boundary energy barrier, or as a result of doping of F as the FZO film showed high mobility.

This work showed that Mo and F are effective dopants and that doping Mo and/or F in ZnO via AACVD greatly improves the conductivity of the undoped ZnO. The MFZO films deposited at 550 °C with 6.2 atom% Mo and 3.6 atom% F showed the lowest resistivity of 5.08 × 10^–3^ Ω·cm, which was 10^5^ orders of magnitude lower in total resistivity compared to the undoped ZnO film and slightly lower than that of the MFZO film deposited via spray pyrolysis.^42^ In addition, in comparison to different types of recently reported codoped ZnO films, the MFZO film with the minimum resistivity has a higher mobility (21.78 cm^2^ V^–1^ s^–1^) than that of Mo/Al-codoped ZnO (8.7 cm^2^ V^–1^ s^–1^),^44^ Al/Ga-codoped ZnO (7.9 cm^2^ V^–1^ s^–1^),^10^ Al/In-codoped ZnO (6.8 cm^2^ V^–1^ s^–1^),^10^ Ga/In-codoped ZnO (3.3 cm^2^ V^–1^ s^–1^),^10^ Ti/Al-codoped ZnO (8.8 cm^2^ V^–1^ s^–1^),^20^ Ti/F-codoped ZnO (14.4 cm^2^ V^–1^ s^–1^),^49^ and Al/F-codoped ZnO (9.7 cm^2^ V^–1^ s^–1^).^6^ Current studies often achieve highly conductive ZnO-based films via high carrier concentration; however, low mobility has limited their applications. This work attempts to further enhance the conductivity by improving the mobility through codoping Mo with F and raising the deposition temperature, which is key to achieve ideal TCOs. The proposed codoped films have the potential for future TCO applications.

The resistivity of the MZO and FZO films reported here was higher than that obtained by using the same AACVD deposition route. Zhao and Ponja et al. reported the resistivity of the MZO and FZO films to be 2.6 × 10^–3^ Ω·cm and 3.02 × 10^–3^ Ω·cm, respectively.^1,6^ Unlike the present study, neither of these studies used the oxygen-rich source (Zn(OAc)_2_·2H_2_O), but instead used the oxygen-poor source (ZnEt_3_) as the host precursor, which resulted in higher carrier concentrations (∼10^20^ cm^–3^) and thus lower resistivities. For the cation-doped ZnO films, the reason that ZnEt_3_ gave such high carrier concentration was due to less charge self-compensation in the lattice, confirmed by the computational study of Demchenko et al.^51^ For the anion-doped ZnO film, the relatively low carrier concentration reported here could be F occupying the oxygen vacancies or interstitial positions in the lattice, both of which accepted an electron, thus reducing the carrier concentration.^43,52^ However, using Zn(OAc)_2_·2H_2_O as the Zn precursor is more advantageous than ZnEt_3_, as ZnEt_3_ is highly pyrophoric and dangerous at both laboratory and industry scales.^15^ Conversely, Zn(OAc)_2_·2H_2_O has low-cost, low-toxicity, air stability and is easy to handle, making it more promising for laboratory and industrial use.^31^ In addition, the resistivity of the above-doped films (10^–3^ Ω·cm) reported in the literature did not improve significantly compared to the undoped ZnO film (10^–3^ Ω·cm), keeping the same order of magnitude. In contrast, the resistivity achieved in this study was noticeably reduced by 1 × 10^3^–10^4^ orders of magnitude.

As for related films deposited via other deposition routes, the resistivity of MZO film prepared by the RF sputtering method was 1.1 × 10^–2^ Ω·cm and was further reduced to 9.5 × 10^–3^ Ω·cm after hydrogen annealing.^34^ The resistivity was lower when deposited via a DC reactive magnetron sputtering method, at 7.9 × 10^–4^ Ω·cm.^35^ Wang et al. reported a resistivity of the FZO film via RF magnetron sputtering of 5.27 × 10^–4^ Ω·cm,^53^ whereas Dineshbabu et al. reported a resistivity of an MFZO film after vacuum-annealed of 1.364 × 10^–3^ Ω·cm.^52^ These results suggested that the electrical performance of the ZnO-based films was also influenced by deposition methods. It is worth mentioning that thin films prepared using PVD techniques (e.g., magnetron sputtering) and the annealing process are costly. Instead, AACVD is a simple, cheap, scalable, and sustainable thin-film preparation method.^37,38^

### 3.9 Optical Properties

**Figure 8:**
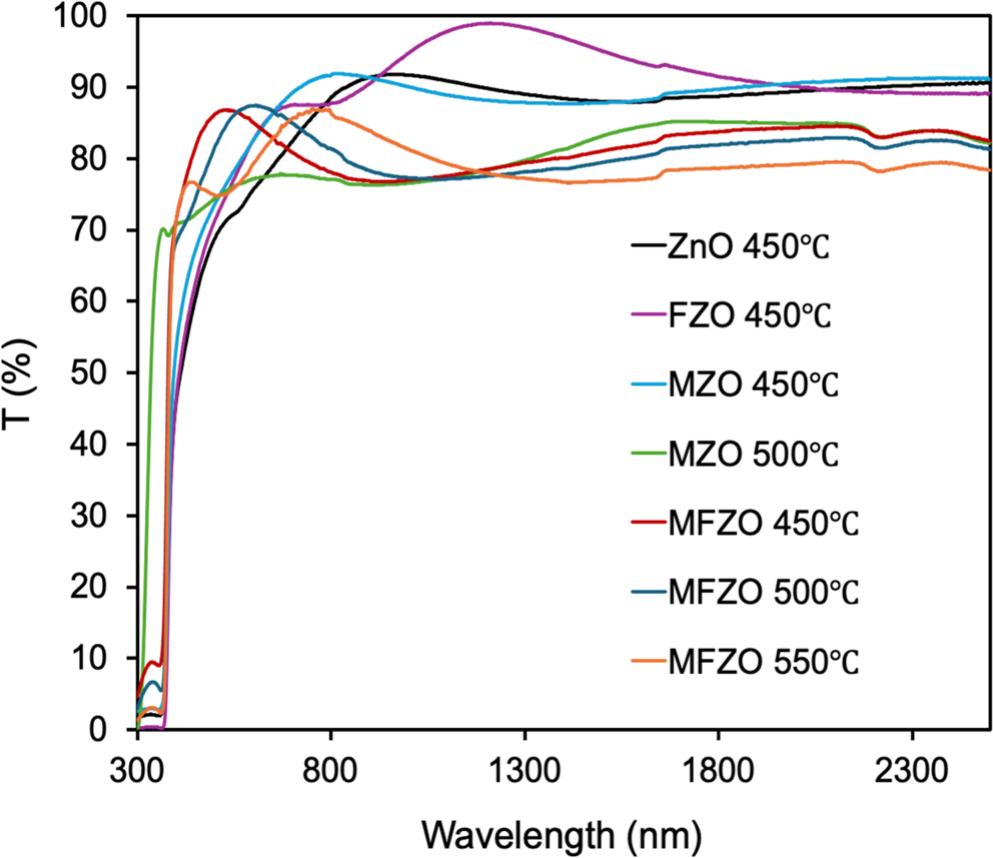
Transmittance spectra of the ZnO, FZO MZO, and MFZO films.

Figure 8 displays the transmittance spectra of the ZnO, MZO, FZO and MFZO films, and the average transmittance of the films across the visible region (400–700 nm) is summarized in Table 4. Among them, the ZnO film showed the lowest transmittance (72%) between 400 and 700 nm. The average transmittance of the FZO, MZO and MFZO films was improved to 75–85%, and some exceeded 80%, meeting the industrial requirements and being comparable to films grown via AACVD,^1,6,54^ as well as other deposition routes such as spray pyrolysis,^42,52^ sol–gel,^33^ and magnetron sputtering.^14^ These findings indicated that Mo and F were effective dopants for improving the optical transparency of the ZnO film.

The reflectance of all films was around 20% at all wavelengths (Supporting Information). The reflectance spectra also showed some interference features in the visible region. This was attributed to the reflections at the three boundaries: air, film, and barrier glass substrate.^10,15,18^ None of the doped and codoped films displayed a decrease in transmittance or an increase in reflectance in the IR region, eventually crossing over, i.e., the plasma edge. This result was quite different from Ga-doped ZnO, Al-doped ZnO, W-doped SnO, and Al/F-codoped ZnO films, where plasma edges can be observed in the transmittance and reflectance spectra.^6,11,15,27^ Therefore, the MZO, FZO and MFZO films were not suitable for low-emission applications that need high reflectivity in the IR region, such as heat mirrors.^6,37,40^

**Figure 9:**
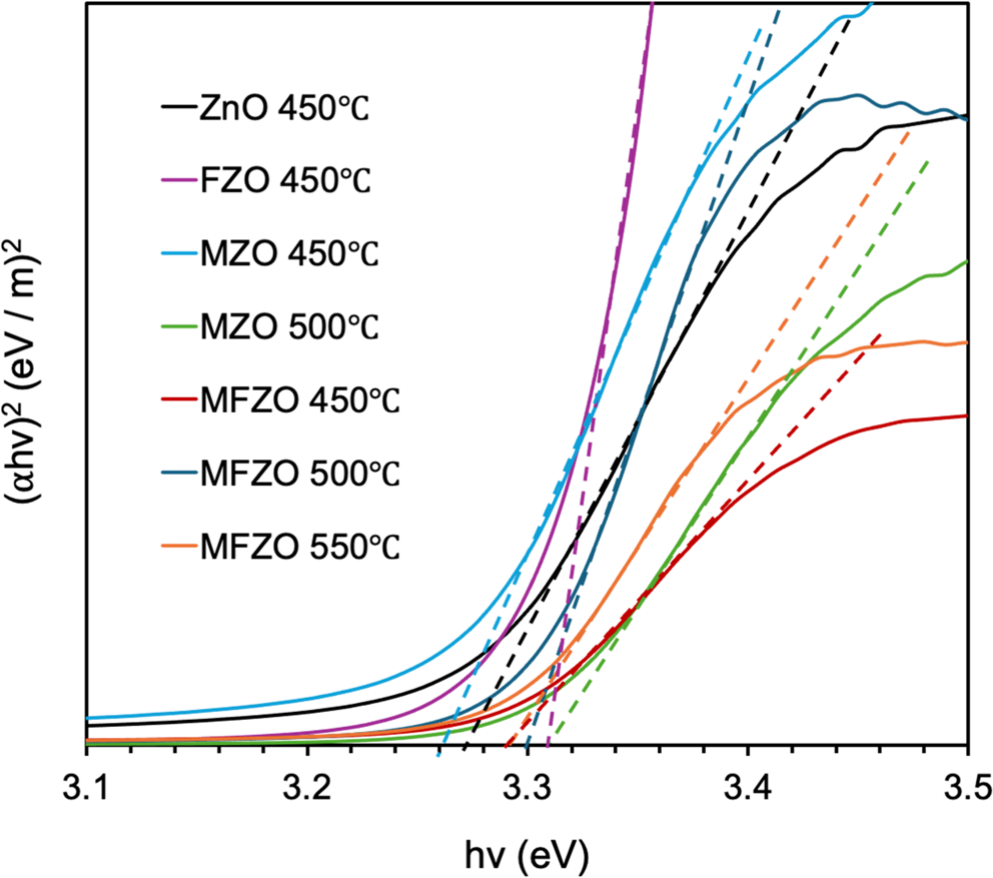
Tauc plots of the ZnO, FZO, MZO, and MFZO films.

To quantitatively assess the optical properties after doping, the bandgap of each film was determined by Tauc plots (Figure 9), (α*h*v)^2^ vs *h*v, and the results are summarized in Table 4. Here, α is the absorption coefficient, *h* is Planck constant, and *v* is photon frequency, and the bandgap was obtained by linear extrapolation of the deepest gradient to the *x*-intercept.^7^ The ZnO film showed a bandgap of 3.27 eV, which agreed with the literature value.^17^ However, the bandgap of the MZO, FZO and MFZO films (∼3.30 eV) did not show a significant change compared to the undoped ZnO film, but a slight increase can be observed. This finding could be interpreted by two incompatible effects. Based on the Mos*sec*-Burstein effect, upon doping, the lower states of the conduction band would be filled with increasing electrons contributed by the dopant, moving the Fermi level into the conduction band, thereby widening the bandgap.^3,5,8^ However, increasing the carrier concentration by the dopant resulted in an increase in charge interactions in the lattice. This would generate many-body effects (e.g., electron–electron scattering) that led to renormalization of the fundamental bandgap of ZnO, eventually leading to bandgap narrowing.^2,29−31,37,55^ In this study, the small variation in the bandgap between the pure and doped/codoped ZnO films indicated that the bandgap widening and narrowing effects were coexistent.

## 4 Conclusions

Transparent and conducting Mo- and F-doped ZnO and Mo/F-codoped ZnO films have been successfully prepared via AACVD, using air-stable and inexpensive precursors. The successful doping of Mo and F in ZnO was confirmed by XPS. XRD only showed hexagonal wurtzite reflections of ZnO upon doping with Mo and/or F but with a slight contraction of the unit cell parameters due to the smaller size of dopants. SEM showed that all of the films had well-defined structures. This study revealed that Mo and F are effective dopants and that doping with Mo and/or F in ZnO via AACVD greatly improves the optoelectronic properties of the pure ZnO film. The visible light transmittance of the doped and codoped films was improved to 75–85%, some exceeded 80%, meeting the industrial requirements. Hall effect measurements showed that codoping of ZnO with 6.2 atom% Mo and 3.6 atom% deposited at 550 °C gave rise to a minimum resistance of 5.084 × 10^–3^ Ω·cm, with free carrier concentration of 5.483 × 10^19^ cm^–3^ and mobility of 21.78 cm^2^ V^–1^ s^–1^. Current studies often achieve highly conductive ZnO-based films with a high carrier concentration but low mobility. This work attempts to increase the mobility through codoping and increasing the deposition temperature to further improve the electrical conductivity, which is key to achieving ideal TCO materials. This is the first time that MFZO films have been synthesized via AACVD, and it also demonstrates that AACVD is a reliable synthesis technique for generating high-quality TCO films.

## Supporting Information Available

Table detailing the range of dopant levels (mol%) studied and reflectance spectra for all of the films (PDF)
