# Supplementary material for: Synthesis and Characterization of Optically Transparent and Electrically Conductive Mo-Doped ZnO, F-Doped ZnO, and Mo/F-Codoped ZnO Thin Films via Aerosol-Assisted Chemical Vapor Deposition
Source: Cryst Growth Des. 2024 Dec 4;24(24):10256–66. doi: 10.1021/acs.cgd.4c01238 (PMC11660145; doi:10.1021/acs.cgd.4c01238)
Supplement: Supplementary file 1 — cg4c01238_si_001.pdf [file cg4c01238_si_001.pdf]

Synthesis and Characterization of Optically Transparent and Electrically Conductive Mo-doped ZnO, F-doped ZnO and Mo/F Co-doped ZnO Thin Films via Aerosol Assisted Chemical Vapour Deposition

Nan Chen[a], Iqra Ramzan[a], Shuhui Li[a], and Claire J. Carmalt[a]*

[a]Materials Chemistry Center, Department of Chemistry, University College London, 20 Gordon Street, London WC1H 0AJ, UK.

Supporting Information

Table S1 The optoelectronic parameters of ZnO, MZO, FZO and MFZO films.

Film	Temperature (°C)	$T_{\lambda 400\text{-}700}$ (%)	E_g (eV)	N (cm^{-3})	μ ($cm^2\ V^{-1}\ s^{-1}$)	ρ (Ω cm)
Undoped ZnO	450	72	3.27	2.94×10^{14}	12.13	9.53×10^{2}
1 mol% Mo	450	86	3.30	1.03×10^{16}	20.64	2.93×10^{1}
2 mol% Mo	450	82	3.29	4.38×10^{17}	7.56	1.89×10^{0}
2.5 mol% Mo	450	82	3.32	3.88×10^{18}	2.48	6.44×10^{-1}
3 mol% Mo	450	77	3.26	8.61×10^{18}	4.01	1.81×10^{-1}
3.5 mol% Mo	450	75	3.28	3.20×10^{18}	1.97	1.88×10^{0}
4 mol% Mo	450	77	3.30	2.62×10^{18}	1.07	3.63×10^{0}
3 mol% Mo	350	92	3.35	5.12×10^{14}	26.42	4.61×10^{2}
3 mol% Mo	400	85	3.31	1.22×10^{17}	3.83	1.33×10^{1}
3 mol% Mo	500	75	3.31	1.25×10^{19}	7.94	6.30×10^{-2}
3 mol% Mo	550	33	3.24	1.59×10^{20}	0.15	2.59×10^{-1}
0.5 mol% F	450	90	3.30	6.93×10^{16}	14.73	6.12×10^{0}
1 mol% F	450	85	3.31	5.41×10^{17}	25.54	4.52×10^{-1}
1.5 mol% F	450	87	3.32	2.81×10^{16}	24.49	9.08×10^{0}
2 mol% F	450	91	3.29	3.44×10^{16}	7.01	2.59×10^{1}
3 mol% F	450	89	3.29	1.03×10^{17}	28.82	2.10×10^{0}
4 mol% F	450	92	3.30	2.91×10^{17}	6.21	3.46×10^{0}
2.1 mol% Mo + 1 mol% F	500	84	3.29	1.08×10^{19}	1.95	2.90×10^{-1}
2.2 mol% Mo + 1 mol% F	500	84	3.29	2.91×10^{19}	1.08	1.50×10^{-1}
2.3 mol% Mo + 1 mol% F	500	83	3.29	6.76×10^{18}	14.93	6.18×10^{-2}
2.4 mol% Mo + 1 mol% F	500	82	3.29	6.19×10^{18}	13.97	7.51×10^{-2}
2.5 mol% Mo + 1 mol% F	500	82	3.30	1.58×10^{18}	21.30	1.90×10^{-1}
2.75 mol% Mo +1 mol% F	500	80	3.28	2.47×10^{18}	11.47	2.20×10^{-1}
3 mol% Mo + 1 mol% F	500	79	3.34	1.39×10^{18}	7.77	5.78×10^{-1}
2.3 mol% Mo + 0.5 mol% F	500	82	3.29	1.25×10^{19}	10.72	5.20×10^{-2}
2.3 mol% Mo + 0.75 mol% F	500	79	3.30	2.58×10^{19}	16.79	2.51×10^{-2}
2.3 mol% Mo + 1 mol% F	500	83	3.29	6.76×10^{18}	14.93	6.18×10^{-2}
2.3 mol% Mo + 1.25 mol% F	500	79	3.28	3.96×10^{18}	6.86	2.30×10^{-1}
2.3 mol% Mo + 1.5 mol% F	500	82	3.28	3.34×10^{18}	4.99	3.74×10^{-1}
2.3 mol% Mo + 0.75 mol% F	450	83	3.29	1.18×10^{19}	8.68	6.10×10^{-2}
2.3 mol% Mo + 0.75 mol% F	500	82	3.30	2.58×10^{19}	16.79	2.51×10^{-2}
2.3 mol% Mo + 0.75 mol% F	550	79	3.29	5.48×10^{19}	21.78	5.08×10^{-3}

$T_{\lambda 400-700}$: average transmittance between 400 – 700 nm; E_g: bandgap; N: carrier concentration/density; μ: carrier mobility; ρ: resistivity.

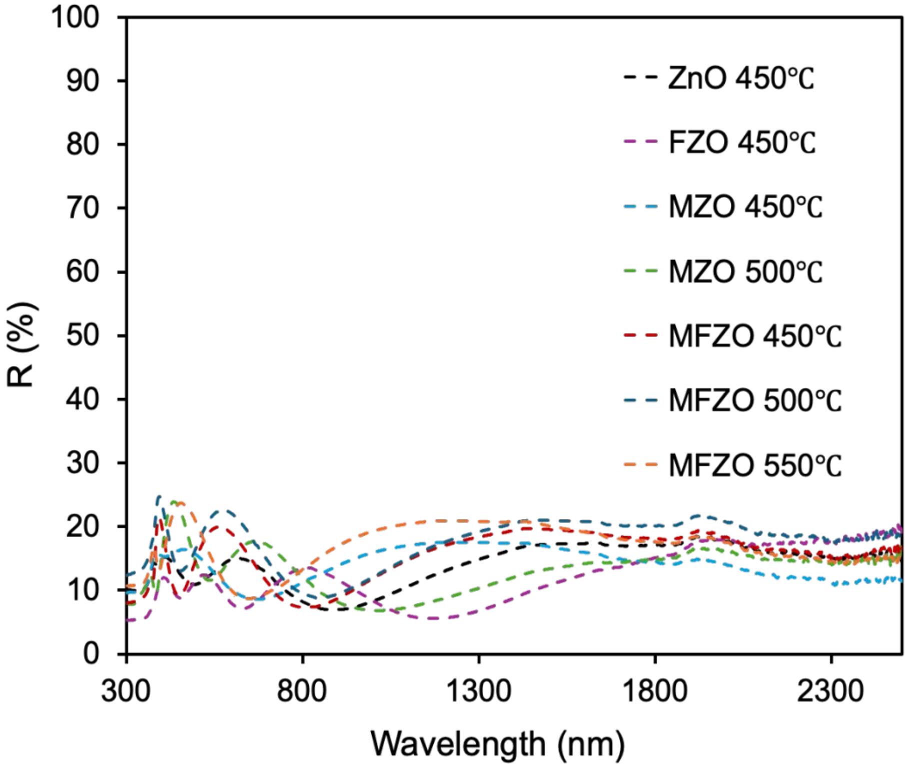

Fig.S1 Reflectance spectra of ZnO, FZO MZO and MFZO films.
